# Importance of Penile Vascularization in Live Donor Penile Transplantation

**DOI:** 10.1590/S1677-5538.IBJU.2025.0195.1

**Published:** 2025-06-20

**Authors:** Luciano A. Favorito

**Affiliations:** 1 Universidade do Estado do Rio de Janeiro Unidade de Pesquisa Urogenital Rio de Janeiro RJ Brasil Unidade de Pesquisa Urogenital - Universidade do Estado do Rio de Janeiro - Uerj, Rio de Janeiro, RJ, Brasil; 2 Hospital Federal da Lagoa Serviço de Urologia Rio de Janeiro RJ Brasil Serviço de Urologia, Hospital Federal da Lagoa, Rio de Janeiro, RJ, Brasil

## Comment

The paper by Djordjevic and colleagues ( 1 ) is very interesting and shows the possibility of using remaining penile tissue, such as preserved corpora cavernosa, the remaining glans tissue with neurovascular components, and the anterior urethra, after feminizing gender affirmation surgery, for live donor penile transplantation. It confirms the technical feasibility and the possibilities of using all remaining penile tissue for possible live donor penile transplantation.

Knowledge of penile anatomy is a crucial step in this procedure. The penis is irrigated by two internal pudendal arteries, branches of the internal iliac (hypogastric) artery. After its various perineal branches, the pudendal arteries combine to form the so-called common penile artery, which divides into three branches: the bulbourethral artery, the dorsal penile artery, and the cavernosal artery. The cavernosal artery is located inside the corpus cavernosum, the bulbourethral artery is responsible for irrigating the corpus spongiosum and urethra, and the dorsal penile artery is located between the tunica albuginea and Buck's fascia ( 2 ). The collateral communications between the bulbourethral artery and the dorsal artery are fundamental for dissection during the procedure described in this paper. This is an example of the importance of anatomy in urological surgery.
